# Direct-current generators based on conductive polymers for self-powered flexible devices

**DOI:** 10.1038/s41598-021-99447-x

**Published:** 2021-10-12

**Authors:** Yanfang Meng, Long Zhang, Guangyuan Xu, Heling Wang

**Affiliations:** 1 Department of Engineering Mechanics, Tsinghua University, Beijing, 100084 China; 2 Center for Flexible Electronics Technology, Tsinghua University, Beijing, 100084 China; 3 Department of Chemistry, Center of Basic Molecular Science, Tsinghua University, Beijing, 100084 China

**Keywords:** Materials for devices, Soft materials

## Abstract

Direct-current generators, especially those based on the Schottky contacts between conductive polymers and metal electrodes, are efficient in converting mechanical stimuli into electrical energy. In contrast to triboelectric and piezoelectric generators, direct-current generators readily produce direct-current outputs and high currents that are crucial for integrating multiple energy-harvesting units in large scale and driving some types of devices. We are focusing on the relationship between Schottky barrier height and performance, systematically investigating the effects of various conductive polymers and electrodes on the outputs by both theoretical simulation and experiments. Tailoring the Schottky barrier height between conductive polymers and metal electrodes is demonstrated a significant approach to design the new DC generators. The preparation method of electrochemical deposition endows the generators flexibility, the linear relationship of current/voltage output vs. strain applied on the generators, combined with the large outputs offer advantages for the generator to work as flexible sensors. Furthermore, a mechanosensation-active matrix array based on direct-current generator for the strain monitoring demonstrated its promising prospects in flexible electronics. The direct-current generators with improved performance could serve as a stream new blood for versatile sensory systems and human–machine interactive interfaces.

## Introduction

To solve the energy crisis, the approach of converting ambient mechanical energy into electrical energy is widely recognized for sustainable power source^1–6^. Self-powered devices that incorporate both energy-harvesting units^7–12^ and functional units (such as sensors)^13–15^ are of great interest in electronic devices^16–18^, photovoltaic devices^19,20^, photoelectric devices^21,22^ and daily uses^23^. To enable the large-scale integration of multiple energy harvesting units to enhance the efficiency, the direct-current output can be recognized as an ideal alternative as it avoids the phase mismatch among separate devices. The triboelectric nanogenerators (TENG) and piezoelectric nanogenerators (PENG) usually require the integration of external rectifying circuits to transform the produced alternating current (AC)^24,25^ to direct current, which largely increases the complexity and reduces the economic efficiency.

Pioneering approaches of generating direct-current outputs from mechanical stimuli included the series-connected p–n junctions bridged by a working electrode proposed by Meng et al*.*^26^ and the ionic polymer-based energy harvester by Hou et al*.*^27^, but the processing technologies involved in those devices were too sophisticated for high-density integrations. Fortunately, Shao et al.^28^ proposed a feasible Au/polypyrrole (PPy)/Al direct-current generator based on the Schottky contact between the conductive polymer (PPy) and the metal electrode (Al). This polymer–metal Schottky contact enables the generator to produce direct-current outputs with long duration. Compared to TENG and PENG, the current output of this generator is also larger by at least 15 times and 20 times, respectively. Lin et al.^29^ also proposed a direct-current generator based on a moving van der Waals Schottky diode. Wang et al. have designed a flexible DC generators based on Au–PPy–Al with output voltage of 3.27 V and current of 329 μA^30^. However, the choice of material in above previous works are limited to certain conductive polymers and metals without widely broadened, while exploring other materials to further increase the current and voltage outputs could benefit the practical application of driving special devices. Apart from PPy and Au/Al, the similar conductive polymers (such as polyaniline (PANI) and polyethoxythiophene (PEDOT)) and other metals also can be incorporated into the direct-current generators and produce similar direct-current output characteristics. However, how to realize much higher current/voltage outputs? In Shao’s et al*.* previous work^28^, the output is closely related to the Schottky barrier height. Tailoring the Schottky barrier height between conductive polymers and metal electrodes is demonstrated a significant approach to maximize the output of generators. Meanwhile, the Schottky barrier height has closely associated with the Lowest Unoccupied Molecular Orbital (LUMO) and Highest occupied Molecular Orbital (HOMO) of the polymers and the work functions of the metals. In this manuscript, to optimize the performance of direct-current generators, we investigate the material combination of various conductive polymers and metals to construct a variety of direct-current generators, and focus on discussing the relationship between the output characteristics of these generators and the Schottky barrier heights. Our work offers an insight for exploring the direct-current generators based on the conductive polymer-metal Schottky contact. The exploration yields a direct-current generator that produces 4 times and 5 times higher current and voltage output under the same mechanical deformation, respectively, than those in previous works. Not only possessing characteristics of direct-current output and higher current, the introduction of electrochemical deposition method during fabrication of direct-current generators renders the devices flexibility, bringing benefit to their application as flexible electronics. Meanwhile, the outputs have nearly the linear relationship with the strain applied on the generator. Therefore, besides energy-harvesting, our generators can serve as self-powered flexible strain sensors as well. In all, our direct-current generator offers a significant insight for designing high-efficient direct-current generators.

## Results

### Basic structures of direct-current generators and properties of the conductive polymers

**Figure 1:**
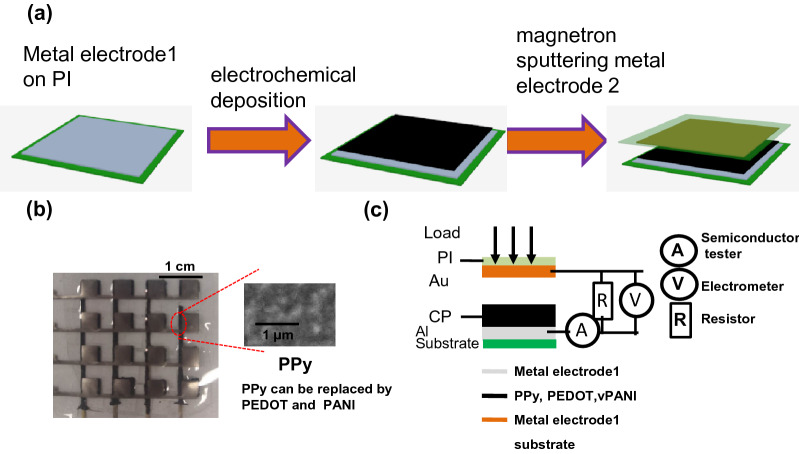
(**a**) Fabrication process of the direct-current generator. (**b**) Photo images of direct-current generator based on conductive polymers. (**c**) Circuit diagram of the direct-current generator.

Basic structures of all the direct-current generators adopted a metal/conductive polymer/metal sandwich geometry. Typical conductive polymers, i.e. polypyrrole (PPy), polyaniline (PANI) and polyethoxythiophene (PEDOT), and various metals were selected to construct the generators based on the principal of the polymer–metal contact conditions. To overcome the inflexibility of previous direct-current generators, such as the Al/PPy/Au generator in Shao et al*.*^28^, a method of electrochemical deposition was proposed to prepare conductive polymers on a flexible substrate coated with metal electrodes film to realize the flexibility. The fabrication procedure is shown in Fig. 1a. Taking Au/PPy/Al generator as an example, firstly, metal electrode film of Cr/Au was magnetron sputtered on polyimide (PI) prior to electrochemically depositing conductive polymer of PPy and metal-coated PI was exposed to UV-Ultraviolet ozone for a treatment to improve surface property. The PI decorated conductive polymer was assembled with another piece of PI with metal film on it.

As shown in Fig. 1b, the photographic images of three kinds of generators, i.e. Au/PPy/Al, Au/PEDOT/Al and Au/PANI/Al, demonstrate their good conformability. The corresponding circuit diagram is systematically illustrated in Fig. 1c. As shown in Fig. 1c, the working principle of the direct-current generators based on the conductive polymer-metal Schottky contact was that output voltage and current stem from deformation of conductive polymers under the external loadings. In our previous work^31^, the energy conversion process when a static compression is presented is discussed. When a direct generator posed with a fixed loading, the thickness of PPy generally varies with time and lasts for 1–2 min.

Different conductive polymers on PI were characterized by the X-ray diffraction (XRD) and scanning electron microscope (SEM) images. Figure S1 illustrate the XRD spectra of PPy (left panel), PEDOT (middle panel) and PANI (right panel). The characteristic diffraction peak of PPy, PEDOT and PANI film were at 26°, 26°, 26° (the diffraction peak corresponds to C (002)). The similar values of XRD demonstrated that the C (002) common crystal faces exist in PPy, PEDOT and PANI. As shown in Fig. S2, the smooth appearances in SEM images of PPy (left panel), PEDOT (middle panel) and PANI (right panel) suggested our conductive polymer films possess good morphology. Owing to that the growth style of electrochemical was modest stationarity, smooth morphologies of these conductive polymers were achieved.

### The output properties of the polymer-metal-Schottky-contact-based direct-current generators

Inspired by the Au/PPy/Al generator in Shao’s^28^ previous works, to produce direct-current output, one metal layer should form an Ohmic contact with the conductive polymer, while the other should form a Schottky contact. As the HOMO of the PPy^32^, PEDOT^33^ and PANI^34^ were − 5.6 eV, − 5.2 eV and − 4.84 eV, respectively, the contacts between PPy and Au, PEDOT and Au, PANI and Cu are Ohmic contacts. Thus, direct-current generators based on Au/PPy/Al, Au/PEDOT/Al and Cu/PANI/Al were designed.

**Figure 2:**
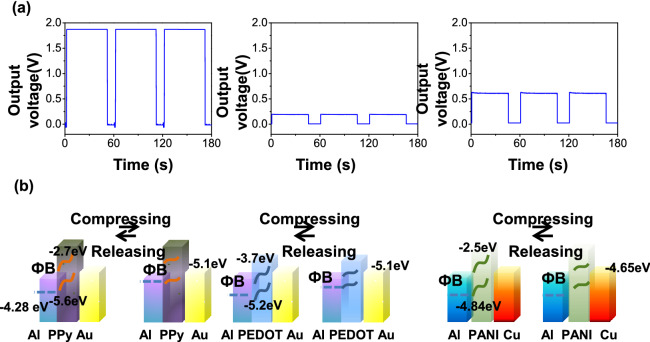
(**a**) Output voltage of direct-current generators measured by oscilloscopes, left panel: Al/PPy/Au, middle panel: Al/PEDOT/Au, right panel: Al/PANI/Cu. (**b**) Proposed energy band diagram of the direct-current generators at freestanding state and at compressing state, left panel: Al/PPy/Au, middle panel: Al/PEDOT/Au, right panel: Al/PANI/Cu.

As shown in Fig. 2a, all the direct-current generators based on Au/PPy/Al (left panel), Au/PEDOT/Al (middle panel), and Cu/PANI/Al (right panel) produce direct-current outputs with long duration, as measured by the oscilloscope. Furthermore, the distinction magnitude of output voltages (1.85 V, 0.2 V and 0.6 V for Au/PPy/Al, Au/PEDOT/Al and Cu/PANI/Al, respectively) of the three generators suggests a clear association of the output on both the conductive polymers and the contact metals. Figure 2b schematically illustrates the energy band diagrams of Au/PPy/Al, Au/PEDOT/Al and Cu/PANI/Al. According to the LUMO and the HOMO of the PPy, PEDOT, PANI and the work functions of metal (Au: 5.1 eV, Cu: 4.65 eV, Al: 4.28 eV and Ag 4.26 eV), the contact between Au and PPy, the contact between Au and PEDOT, are Ohmic contact. the contact between Al and PPy, the contact between Ag and PEDOT, and the contact between Al and PANI are Schottky contact. The contact between Cu and PANI is also Schottky contact. Due to the HOMO of PANI is − 4.84, the work functions of metal (Au: 5.1 eV, Cu: 4.65 eV, Al: 4.28 eV and Ag 4.26 eV), all the metal form Schottky contact with PANI. However, the work function of Cu is approximate to the PANI. So, although all the metal form Schottky contact with PANI, the contact between Cu and PANI is approximate Ohmic contact. To confirm the contact condition between conductive polymer and metal electrode, *I*–*V* measures for all different contacts is carried out (as shown in Fig. SI4). Figure SI3a depicts all the contact: the contact between (Au–PPy) is Ohmic contact, the contact between (Ag–PPy), the contact between (Al–PPy), the contact between (Cu–PPy) and the contact between (Pt–PPy) are Schottky contact. Figure SI3b indicates that the contact between (Au-PEDOT) is Ohmic contact and the contact between (Ag-PEDOT), the contact between (Al-PANI), the contact between (Cu-PEDOT) and the contact between (Pt-PEDOT) are Schottky contact. Figure SI3c shows all the contact: the contact between (Au-PANI), the contact between (Ag-PANI), the contact between (Al-PANI), the contact between (Cu-PANI) and the contact between (Pt-PANI) are Schottky contact. However, the contact between Cu and PANI is relatively approximate Ohmic contact.

As shown in That is, for a direct-current generator with metal/conductive polymers/metal configuration, one metal forms Ohmic contact with the conductive polymer and the other metal forms Schottky contact with the conductive polymer^34,35^.

This energy band diagram illustrates the mechanism underlying the direct-current generator. The direct-current output origins from the mechanical-induced interfacial charge accumulation and the energy-band bending. As a result, the Schottky barrier between the conductive polymer and the metal that forms the Schottky contact reduces and drives the movement of electrons from conductive polymers to the other metal electrodes^36–40^.

In our previous work^31^, the mechanism of the direct-current output based on Au/PPy/Al generator was investigated. The current density involved is given as:

1 $$J_{0} = A^{*} T^{2} e^{{\frac{ - \phi }{{kT}}}}$$

where, A* is the effective Richardson’s constant, *Φ* is the effective barrier potential, *T* is the absolute temperature, *k* is the Boltzmann constant. The barrier potential under deformation can be thus estimated based on Eq. (1).

According to the above equation, the output currents under the external loadings are associated with the Schottky barrier height between the metal and the conductive polymer that forms the Schottky contact, which is equivalent to that the HOMO value of the conductive polymer subtracts the work function of the contacted metal. Do the LUMO and HOMO of the conductive polymer also vary if the conductive polymers experience deformation? Computational simulations are conducted to address this question, as presented below.

### Computation study on the HOMO and LUMO of the conductive polymers to reveal the mechanism

**Figure 3:**
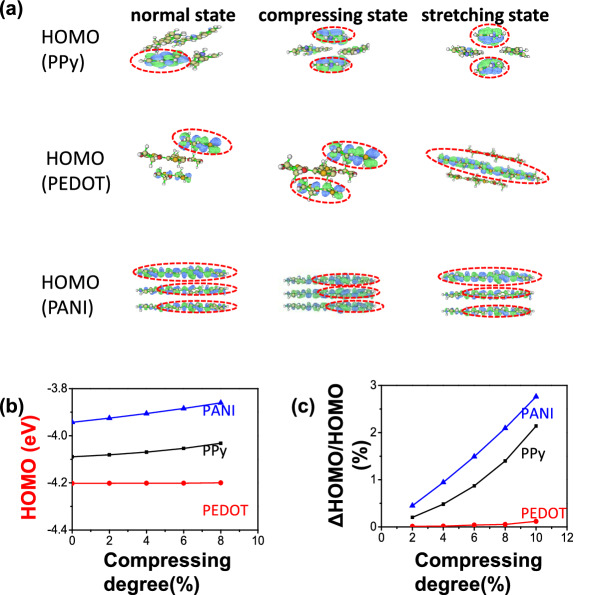
(**a**) The electronic densities distributions of HOMO of PPy (top panel), PEDOT (middle panel) and PANI (bottom panel) in normal state (left panel), compressing state (middle panel) and stretching state (right panel). (**b**) HOMO vs. deformations (2%, 4%, 6%, 8%,10%) for conductive polymer PPy, PEDOT and PANI. (**c**) The relative change in HOMO of PPy, PEDOT and PANI under deformations.

Figure 3a shows the HOMO of PPy (top panel), PEDOT (middle panel) and PANI (bottom panel) crystal fragments in normal state (left panel), compressing state (4% deformation, middle panel) and stretching state (4% deformation, right panel), respectively. As shown in the left panel of Fig. 3a, the electronic densities of the HOMO of the PPy in normal state are predominantly localized on one layer of pyridine ring. In the compressing state (4% deformation) and in the stretching state, the electronic densities of the HOMO are predominantly localized on part of pyridine rings in two different layers. That is, the electronic densities of the HOMO of the PPy crystal fragments change with their deformations. The distributions of the electronic densities of the HOMO change in PEDOT (middle panel) and PANI (bottom panel) under the compressing state and increase distance state, as well.

Specially, as the bandgap of PANI is sensitive to the dopants^41–43^, acetic acid that served as the dopant was added into the well-prepared PANI film to alter the bandgap. The bandgap (*E*_g_) of PANI between HOMO and LUMO is calculated to be *E*_g_ = *h*c/λ. Where, *h* is the Planck’s constant, *c* is the light velocity in vaccum, and λ is the wavelength in the UV–Vis absorb spectrum of PANI (as shown in Fig. SI4a). It can be calculated that the *E*_g_ of PANI before being doped and after being doped are 2.57 eV and 2.84 eV, respectively. The HOMO of PANI between HOMO and LUMO is calculated according to the equation: *E*_HOMO_ = − |eE_1/2_ + 4.5 eV + *E*_SCE_|, where, *E*_SCE_ is standard electrode potential of the reference electrode. The e*E*_1/2_ can be extracted from the volt-ampere characteristics (*CV*) test (as shown in Fig. SI4b). It can be calculated that the HOMO of PANI before being doped and after being doped are − 4.84 eV and − 5.24 eV, respectively. Do to  −*E*_LUMO_ = −*E*_g_ − *E*_HOMO_, the LUMO of PANI before being doped and after being doped are − 2.27 eV and − 2.4 eV, respectively. Therefore, doping can influence the bandgap (Eg) and HOMO of PANI and further affects the output of the DC generator based on PANI.

The corresponding LUMO of the conductive polymers in their normal state, compressing state (4% deformation) and stretching state (4% deformation) are also studied (illustrated in Fig. SI5a), showing a clear distinction similar to the HOMOs. The changes of LUMO and HOMO of conductive polymers in deformed state (compressing or stretching) indicate that the output may be related to the variation of frontier molecular orbital of conductive polymers.

The mechanical-induced deformation changes not only the electronic densities, but also the values of LUMO and HOMO of conductive polymers. The calculated HOMO of the conductive polymer: PPy, PEDOT and PANI are shown in Fig. 3b. As shown by the blank line in Fig. 3b, the 2%, 4%, 6%, 8% and 10% deformation can change the HOMO (ΔHOMO/HOMO) of PPy crystal fragments relatively by 0.204%, 0.484%, 0.871%, 1.400% and 2.140%, respectively. (The Schottky barrier values are closely related to the HOMO values of conductive polymers and work functions of contacted electrodes). For PEDOT, under the same 2%, 4%, 6%, 8% and 10% deformations, the relative changes in HOMO are 0.013%, 0.016%, 0.041%, 0.118% and 0.21%, respectively (blue line in Fig. 3b). For PANI, the relative changes are 0.451%, 0.946%, 1.491%, 2.094% and 2.763%, respectively (red line in Fig. 3b). Figure 3c compares the relative change in HOMO *vs.* compression curves of PANI, PPy, PEDOT, respectively. Under the same deformation, PANI clearly exhibits a much larger change in HOMO than PPy and PEDOT. Recalling that the direct-current generator that involves PANI also shows a larger voltage output, the analysis indicates an association of the output to the HOMO of the conductive polymers. Similar, LUMO vs. deformations (2%, 4%, 6%, 8% and 10%) for conductive polymer PPy, PEDOT and PANI illustrate in Fig. SI5b.

### The voltage/current output of direct-current generators with different conductive polymers and different electrodes

To realize the optimized output of the direct-current generators, it is imperative to make clear what is the leading factor related to the magnitude of the output. To this aim, three factors: conductive polymers, the contacted electrodes form the Ohmic contact and the contacted electrodes form the Schottky contact are discussed respectively.

**Figure 4:**
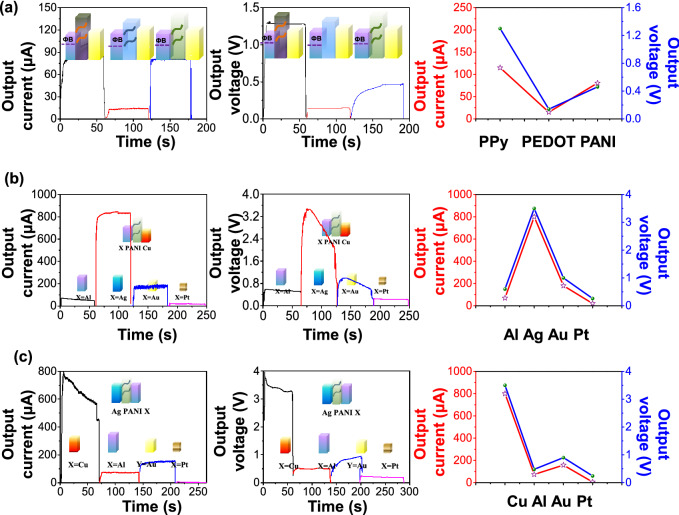
(**a**) Influence of different conductive polymers on direct-current generator. Output currents (left panel) and output voltages (middle panel) of direct-current generators: Au/PPy/Al, Au/PEDOT/Al, Au/PANI/Au (4% compressive deformation), respectively. Right panel: The relationship of the output currents/voltages with varied contacted metals. (**b**) Influence of different Schottky-contacted metals (fixed the Ohmic-contacted metals) on PANI-based direct-current generator. Output currents (left panel) and output voltages (middle panel) of direct-current generators: Cu/PANI/Al, Cu/PANI/Ag, Au/PANI/Cu, Pt/PANI/Cu (4% compressive deformation), respectively. Right panel: the relationship of the output currents/voltages with varied contacted metals. (**c**) Influence of different contacted metals metals (fixed one Schottky-contacted metals) on PANI-based direct-current generator. Output currents (left panel) output voltages (middle panel) of direct-current generators: Ag/PANI/Cu, Ag/PANI/Al, Ag/PANI/Au, Ag/PANI/Pt (4% compressive deformation), respectively. Right panel: The relationship of the output currents/voltages with varied contacted metals.

Figure 4a presents the output currents (left panel) and output voltages (middle panel) of three direct-current generators (Au/PPy/Al, Au/PEDOT/Al and Au/PANI/Al) with the same electrodes but different conductive polymers. Under 4% compressive deformation, the output currents of Au/PPy/Al, Au/PEDOT/Al and Au/PANI/Al are 120 μA, 15 μA and 80.5 μA, respectively as shown in the left panel of Fig. 4a. The output voltages are 1.3 V, 0.14 V and 0.46 V, as shown in the middle panel of Fig. 4a. It is noteworthy that the voltage values of Au/PEDOT/Al and Au/PANI/Al in the Fig. 4a are different from those in Fig. 2a (the voltage values of Au/PPy/Al in Fig. 4a and in Fig. 2a are 1.3 V and 1.85 V, respectively, while, the voltage values of Au/PEDOT/Al in Fig. 4a and in Fig. 2a are 0.14 V and 0.2 V, respectively). The reason for that can be explained by the equipment issue, that is: the voltage measurements in Fig. 4a and in Fig. 2a were by Keysight B1500A Semiconductor Device analyzer and the oscilloscope, respectively.

The relationship of the output currents/voltages with varied conductive polymers are intuitively shown in the right panel of Fig. 4a. The output currents and the output voltages basically show the same dependency on the conductive polymers. The current density under deformation is given as:

2 $$J_{deformation} = A^{ * } T^{2} e^{{\frac{{ - \phi_{deformation} }}{kT}}}$$

According to the equation, where − *Φ*_deformation_ is the Schottky barrier variation after the deformation, the output currents are closely related to the Schottky barrier variation before and after deformation: − *Φ*_*deformation*_ = (*W*_*non-deformation*_ − *HOMO*_*non-deformation*_) − (*W*_*deformation*_ − *LUMO*_*deformation*_) = *HOMO*_*deformation*_ − *LUMO*_*non-deformation*_, that is ΔHOMO (the work functions of the metals are not changed during deformation). Higher ΔHOMO leads to higher *J*_*deformation*_. The relative change of HOMO (ΔHOMO/HOMO) of PPy, PEDOT and PANI under 4% deformation are 0.484%, 0.06% and 0.946%, respectively. This trend agrees with the observation that the current/voltage output of the generator based on PPy is higher than that of PEDOT. However, although in the deformed state the relative change of HOMO of PANI is higher than that of PPy, the output of Au/PANI/Al is lower than that of Au/PPy/Al. It can be explained by the nature of contacts between conductive polymers and metals. In the Au/PANI/Al generator, both metal layers form Schottky contact with the conductive polymer; in the Au/PPy/Al generator, one metal layer (Al) forms Schottky contact while the other (Au) forms Ohmic contact. These results and analysis demonstrate that the output properties are closely related to the HOMOs of conductive polymers under deformation.

To explore the effect of the metal electrodes on the output properties, we construct various generators with the conductive polymer fixed to be PANI. To begin with, one metal layer is also fixed to be Cu with PANI (the contact between Cu and PANI is approximate to the Ohmic contact), and various metals are adopted in the other metal layer that forms Schottky contact. Thus, generators of Cu/PANI/Al, Cu/PANI/Ag, Cu/PANI/Au, Cu/PANI/Pt are investigated. For this Eq. (2), the output currents have close relationship with the − *Φ*_deformation_ = (*W* − *H*_deformation_), where *W* and *H*_*deformation*_ represent the work functions of contacted metal and the HOMO of conductive polymers after deformation, respectively. The values of − *Φ*_*deformation*_(Al-PANI) − *Φ*_*deformation*_(Ag-PANI), − *Φ*_*deformation*_ (Au-PANI) and − *Φ*_*deformation*_(Pt-PANI) are 0.96, 0.98, 0.14 and − 0.41, respectively. The devices: Cu/PANI/Al, Cu/PANI/Ag, Cu/PANI/Au, Cu/PANI/Pt followed the law that: the higher − *Φ*_*deformation*_ (metal-PANI) (relative value, not absolute value), the higher output current. However, the − *Φ*_*deformation*_ (Au-PANI) was not follow the above law. Further work will be conducted to investigate the underlying reason. As shown in the left panel of Fig. 4b, the output currents of the direct-current generators composed of Cu/PANI/Al, Cu/PANI/Ag, Cu/PANI/Au, Cu/PANI/Pt are 69 μA, 800 μA, 180 μA and 20 μA (4% compressive deformation), respectively. As shown in the middle panel of Fig. 4b, output voltages of these direct-current generators are 0.6 V, 3.5 V, 1 V and 0.26 V (4% compressive deformation), respectively. Whatever output currents or output voltages agree very well with the law: higher − *Φ*_*deformation*_ (metal-PANI) leads to the higher output current. It should be emphasized that both the current output (800 μA) and the voltage output (3.5 V) of the Cu/PANI/Ag generator are much higher than those in the previous work of Shao et al*.* (maximum voltage 0.8–0.9 V, maximum current 200 μA). The Cu/PANI/Ag generator stands out of the investigated generators because under deformation the HOMO of PANI changes much more than that of PPy and PEDOT, such that the value of − *Φ*_*deformation*_ (Ag-PANI) is much larger. Meanwhile, this generator also ensures that one metal layer forms Schottky contact with the conductive polymer, while the other layer forms Ohmic contact. The relationship of the output currents/voltages with varied metal electrodes which form Schottky contact with the conductive polymers are intuitively shown in the right panel of Fig. 4b. The variation of the current output and the voltage output show basically the same trend.

The calculated energy conversion efficiency is about 16.8% for Ag/PANI-HAc/Cu generator (“Supporting information”).

To further illustrate the effect of the metal that forms Schottky contact with the conductive polymers, the polymer PANI in the above study is replaced by PEDOT (Fig. SI6) and PPy (Fig. SI7), respectively, while, the other setups are the same. The same trend is found in PANI and PPy: higher − *Φ*_deformation_ (metal-PEDOT/PPy) leads to a higher output current.

In the above study, as well as in Shao’s work, one of the two metal electrodes forms an Ohmic contact with the conductive polymer and the other forms a Schottky contact. Can the generators produce current/voltage outputs if both metal electrodes form the Schottky contacts with the conductive polymer? To answer this question, the direct-current generator based on Ag/PANI/Cu, Ag/PANI/Al, Ag/PANI/Au and Ag/PANI/Pt are prepared. It is worth mentioning that the metal electrodes of Al, Ag and Au form the Schottky contacts with PANI, such that in these generators both metals form Schottky contact. To our surprise, these generators still produce current and voltage outputs under deformation (4% in compressive state), as shown in Fig. 4c. As shown in the middle and right panel of Fig. 4c, though the outputs of the generators with both metal forming the Schottky contact with the conductive polymer are smaller (74 μA, 0.48 V for Ag/PANI/Al; 158 μA, 0.9 V for Ag /PANI/Au; 6 μA, 0.24 V for Ag/PANI/Pt) than the counterpart of the generators with one Ohmic contact and one Schottky contact (800 μA, 3.5 V for Ag/PANI/Au), the current outputs are still higher than those in general triboelectric nanogenerators (below 10 μA).

### The relationship between the voltage/current output of the direct-current generators and the external loadings

Besides energy-harvesting, the direct-current generators studied above can also serve as sensors, as their outputs show clear relationships with the external stimuli. Our previous work^30^ revealed that the output characteristics of the generator based on PPy were closely related to the deformation of the PPy. The mechanism is that the amount of accumulated spatial charges at the PPy/Al electrode interface depends on the degree of deformations, leading to different amount of reduction of the Schottky barrier, and consequently the change in the output current/voltage (according to Eq. 2).

To further illustrate the relationship between the voltage/current output of the direct-current generators and the loadings posing on them, voltage/current vs deformation of the Au/PPy/Al generator is illustrated in Fig. SI8 with the left panel for output current and the right panel for output voltage. The output currents of Au/PPy/Al generator are 40 μA, 89 μA, 150 μA and 170 μA for 2%, 4%, 6% and 8% deformation, respectively, while, the output voltages of the Au/PPy/Al generator are 0.15 V, 0.19 V, 0.42 V and 0.77 V for 2%, 4%, 6% and 8% deformation, respectively. As shown in the right panel of Fig. SI8, the output current/voltage exhibits nearly linear relationship with the deformation. Furthermore, this linearity also hold true for the outputs of the direct-current generators based on other conductive polymers (Ag/PEDOT/Au and Cu/PANI/Ag), as shown in Figs. SI9 and SI10. The linear relationship between current/voltage outputs and strain posed on the generators, combined with the large outputs and flexibility presented in prior sections, demonstrate a great promising of our generators for applications in flexible sensors.

### The flexible direct-current generator matrix array for mechanosensation sensor

Recently, self-powered flexible electronics^44^ integrated with energy harvesting units (i.e. direct-current generators) have arose extensive attention. Due to the limitation of the fabrication in Shao’s previous work, the reported generator was not flexible and therefore not practical to apply to flexible electronics. The electrochemical deposition method adopted here enables an array of direct-current generators based on conductive polymers to be integrated into flexible electronics, via fabricating them on soft elastomeric substrate such as PDMS. The flexibility and stretchability of such device allow the conformal contact with various target objects, which offers a promising avenue to apply as mechanosensation sensor matrix array for applications.

**Figure 5:**
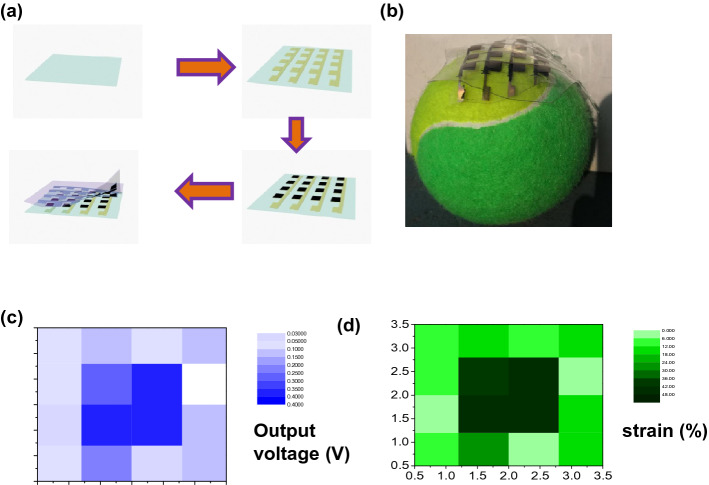
Strain sensing array based on direct-current generators. (**a**) Fabrication process of the array of direct-current generators. (**b**) Photo images of direct-current generator array conformally attached on a tennis ball. (**c**) Mapping of voltage output of every unit in the array. (**d**) Mapping of pressure extracted from the measured voltages.

To demonstrate this application, an array of direct-current generators on a PDMS substrate (4 × 4 pixels in cross-intersected configuration) was fabricated to form a mechanosensation sensor matrix array to sense the strain distribution. The fabrication procedure is illustrated in Fig. 5a, with the process being the same as that for the single unit device in Fig. 1c. The mechanosensation sensor matrix array based on the direct-current generator is feasibly attached on a tennis ball conformally (As shown by the photo image in Fig. 5b). The electrical performance of each unit of the sensor array in the sensation matrix is characterized. As the matrix array is attached on a tennis ball, every unit undergoes a different magnitude of compressing deformation and produces a different voltage signal. The sensing signals are closely related to the level of deformation of the conductive polymers according to the strain exerted on each sensing unit. The output voltages of each unit are measured and plotted in 2D black-and-white coordinates (Fig. 5c). Notably, the circuit design of the matrix guarantees that all the output voltages of the sensing units are read out individually without crosstalk, and meanwhile are recorded simultaneously. The corresponding strains exerted on each unit of the mechanosensation sensor matrix are extracted from the output voltages and shown in Fig. 5d. Only the sensing units have the output signals when the strains are posed on them, suggesting no crosstalk in the mechanosensation matrix.

## Conclusions

As a summarization, for integrating multiple energy-harvesting units in large scale and driving some types of devices, it is highly desirable to take the advantages of direct-current generators to feasibly produce direct-current outputs and high currents with long duration. With the aim to optimizing the output of the direct-current generator based on conductive polymers, various direct-current generators based on PPy, PEDOT and PANI are explored. Particularly, the unparalleled output current and output voltage of the Cu/PANI/Al direct-current generators surpass the previous ones in literature (output current and output voltage reach 800 μA and 3.5 V, respectively). Besides energy-harvesting, our flexible generator also can be applied as strain sensor, as the output current/voltage has nearly the linear relationship with the magnitude of strain applied on the conductive polymers. A mechanosensation-active matrix array based on direct-current generator of conductive polymers is applied in 2D spatial image of the strain distribution and demonstrates its promising prospects in flexible electronics. Overall, our direct-current generator opens a new opportunity for high-efficient flexible versatile devices for both energy-harvesting and sensing. Discussion should be succinct and must not contain subheadings.

## Methods

### Subsection materials and methods

Lithium perchlorate (LiClO_4_) (≥ 99%), pyrrole (≥ 99%), aniline and ethoxythiophene were bought from Sigma-Aldrich.

1. Electrochemical deposited of PPy, PEDOT and PANI.
  To guarantee the absence of air gap between conductive polymer layer and the upper Au electrode, the commercial available PI was made pre-treatment for hyper-planarization, according to the technology for heating processing. Prior to heating treatment at 80–100 °C for 10 min, polyamide acid precursor was being spun at 2000 rpm/min. Then, the polyamide acid precursor-coated PI undergone annealing for several continuous stages: 180 °C for 20–30 min, 200 °C for 10 min, 220 °C for 10–20 min, 250 °C for 10–30 min. Consequently, the obtained PI is super planar.
  7 nm/80 nm Cr/Au or Cr/Pt, Al, Ag, Cu, film was deposited on PI. After UV-ozone treatment of PI, polypyrrole and polyethoxythiophene were grown on metal film from pyrrole and ethoxythiophene monomers through electrochemical deposition in cyclic voltammetry under constant positive potential of 0.7 V and 1 V in 0.1 mol/L lithium perchlorate, respectively. The electrochemical deposited aniline to prepare polyaniline in cyclic voltammetry under constant positive potential of 0.7 V in 0.1 mol/L sodium sulfite. The chemical activation of polypyrrole was performed by heating (at a ramp rate of 3 °C/min, a KOH/PPy mixture (at a KOH/PPy weight ratio of 2 or 4) under a nitrogen flow to a final temperature of between 600 and 850 °C for 1 h. The activated samples were then thoroughly washed with 10 wt% HCl to remove any inorganic salts, and then with distilled water until a neutral pH and then dried in an oven at 120 °C.
  Particularly, due to the band-gap of PANI sensitive to the dopant^40–42^, the dopant: acetic acid was added into the well-prepared PANI film to alter the band-gap.
2. Magnetron sputtering the other metal electrode film and device package.
  7 nm/80 nm Cr/Au, Cr/Pt, Cr/Al, Cr/Ag and Cr/Cu film were deposited on another piece of PI and assembled with PI coated with conductive polymers in a face-to-face fashion. The electric wires were extracted from the Au film and the Al film, respectively.
3. Computational methods.
  DFT methods were applied for the geometric optimization and the orbital stimulation (i.e. the LUMOs, the HOMOs) of the crystal fragments of conductive polymers (PPy, PEDOT and PANI). All of the above computations were conducted in the Gaussian 09 program suite using the functional of Beck’s three-parameter hybrid exchange function with Lee–Yang–Parr gradient-corrected correlation functional (B3LYP) with a generalized gradient approximation (GGA)^45–47^ of Beck’s exchange function. Lanl2dz was chosen as the basis set.

### Characterization

Surface morphology was obtained by a SEM (FEI/Quanta 450 FEG). X-ray diffraction (XRD) patterns were assessed with a Panalytical X-ray diffractometer with a Cu radiation of 2.2. The samples were scanned in the 2*θ* range of 10°–80° with a step size of 0.05. The voltage measurements in Fig. 2 were by oscilloscope and all other electrical performances of all kinds of direct-current generators were measured by a Keysight B1500A Semiconductor Device analyzer with the deformation real-time monitored by the digital display thickness gauge. All measurements were carried out under ambient conditions.

During the electrical character test on the devices under deformation, the initial thickness of the device is recorded and compressed according to the corresponding percentage monitored by the digital display thickness gauge.

## Supplementary Information

Supplementary Figures.
