# Supplementary material for: Direct-current generators based on conductive polymers for self-powered flexible devices
Source: Sci Rep. 2021 Oct 12;11:20258. doi: 10.1038/s41598-021-99447-x (PMC8511334; doi:10.1038/s41598-021-99447-x)
Supplement: Supplementary file 1 — Supplementary Figures. [file 41598_2021_99447_MOESM1_ESM.doc]

**Supporting Information**

**Direct-current generators based on conductive polymers for self-powered flexible devices**

*Yanfang Meng 1,2, *+, Long Zhang 3,+, Guangyuan Xu 1,2, and Heling Wang 2*

^1^ Department of Engineering Mechanics, Tsinghua University, Beijing, 100084,China.

^2^ Center for Flexible Electronics Technology, Tsinghua University, Beijing, 100084, China.

^3^ Center of Basic Molecular Science, Department of Chemistry, Tsinghua University, Beijing, 100084, China

* Corresponding author: [yanaimengmeng@126.com](mailto:yanaimengmeng@126.com)

**Supplementary Text**

**Figure S1**. XRD of synthesized PPy(left panel), PEDOT(middle panel)and PANI(right panel).

**Figure S2**. SEM of synthesized PPy, PEDOT and PANI.

**Figure S3.** *I-V* measurement of conductive polymers and contact metal. Left panel: Au/PPy/Au, Ag/PPy/Ag, Al/PPy/Al, Cu/PPy/Cu, Pt/PPy/Pt; middle panel: Au/PEDOT/Au, Ag/PEDOT/Ag, Al/PEDOT/Al, Cu/PEDOT/Cu, Pt/PEDOT/Pt; right panel: Au/PANI/Au, Ag/PANI/Ag, Al/PANI/Al, Cu/PANI/Cu, Pt/PANI/Pt.

**Figure S4.** Left panel: UV-vis absorb spectrum of PANI before (blank line) and after (blue line) doped. Right panel: The volt-ampere characteristics (*CV*) curves of PANI before (blank line) and after (blue line) doped.

**Figure S5.** (a) The electronic densities distributions of LUMO of PPy (top panel), PEDOT (middle panel) and PANI (bottom panel) in normal state (left panel), compressing state (middle panel) and increase distance state(right panel). (b) Influence of varied deformation (2%, 4%, 6%, 8% and 10%) of conductive polymer PPy, PEDOT and PANI on HOMO

**Figure S6**. Influence of different contacted metals on PEDOT-based direct-current generator. Output currents, output voltages (middle panel) of direct-current generators: Au/PEDOT/Al, Au/PEDOT/Ag, Au/PEDOT/Cu, Au/PEDOT/Pt (4% compressive deformation), respectively. The relationship of the output currents/voltages with varied contacted metals (right panel).

**Figure S7.** Influence of different contacted metals on PPy-based direct-current generator. Output currents (left panel), output voltages (middle panel) of direct-current generators: Au/PPy/Al, Au/PPy/Ag, Au/PPy/Cu, Au/PPy/Pt (4% compressive deformation), respectively.

**Figure S8.** Output characteristics of Al/PPy/Au generator under different deformations. Output current (left panel) and voltage (middle panel) of Al/PPy/Au generator during variation degree of compressing deformation and corresponding extracted the relationships of current ~ compressing deformation and voltage ~ compressing deformation (right panel).

**Figure S9**. Output characteristics of the Ag/PEDOT/Au generator under different deformations. Output current (left panel), voltage (middle panel) of Ag/PEDOT/Au generator during variation degree of compressing deformation and corresponding extracted the relationships of current ~ compressing deformation and voltage ~ compressing deformation (right panel).

**Figure S10**. Output characteristics of Ag/PANI/Cu generator under different deformations. Output current (left panel), voltage (middle panel) of Ag/PANI/Cu generator during variation degree of compressing deformation andcorresponding extracted the relationships of current ~ compressing deformation and voltage ~ compressing deformation (right panel).

**Figure. S1.** XRD of synthesized PPy(left panel), PEDOT(middle panel) and PANI (right panel).

**Figure. S2.** SEM of synthesized PPy, PEDOT and PANI.

**Figure. S3.** *I-V* measurement of conductive polymers and contact metal. Left panel: Au/PPy/Au, Ag/PPy/Ag, Al/PPy/Al, Cu/PPy/Cu, Pt/PPy/Pt; middle panel: Au/PEDOT/Au, Ag/PEDOT/Ag, Al/PEDOT/Al, Cu/PEDOT/Cu, Pt/PEDOT/Pt; right panel: Au/PANI/Au, Ag/PANI/Ag, Al/PANI/Al, Cu/PANI/Cu, Pt/PANI/Pt.

**Figure. S4** Left panel: UV-vis absorb spectrum of PANI before (blank line) and after (blue line) doped.

Right panel: the volt-ampere characteristics (*CV*) curves of PANI before (blank line) and after (blue line) doped.

**Figure. S5** (a) The electronic densities distributions of LUMO of PPy (top panel), PEDOT (middle panel) and PANI (bottom panel) in normal state(left panel), compressing state(middle panel) and increase distance state(right panel).

(b) Influence of varied deformation (2%, 4%, 6%, 8% and 10%) of conductive polymer PPy, PEDOT and PANI on HOMO

Calculation of energy conversion efficiency:

The energy conversion efficiency of the device is calculated according to the following equation:


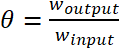
 ……(S1)

The input mechanical energy *W*_input_ per cycle is referred as the absorbed strain energy of the conductive polymer materials.


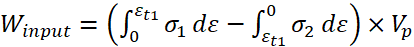
 ……(S2)

This energy was extracted based on the stress-strain curve in the compression-decompression process. Where, *σ*_1_ is the compression stress, *σ*_2_ is the decompression stress, *dε* is the strain value, *V*_p_ is the plate volume.

The electric output energy *W*_output_ is calculated by the equation as following:


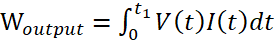
 ……(S3)

Where, *V*(t) and *I*(t) are assigned as real-time voltage and real-time current, respectively.

According to the above equations, energy conversion efficiency is about 16.8% for Ag/PANI-HAc/Cu generator.

**Figure. S6** Influence of different contacted metals on PEDOT-based direct-current generator. Output currents, output voltages (middle panel) of direct-current generators: Au/PEDOT/Al, Au/PEDOT/Ag, Au/PEDOT/Cu, Au/PEDOT/Pt (4% compressive deformation), respectively. The relationship of the output currents/voltages with varied contacted metals (right panel).

**Figure. S7** Influence of different contacted metals on PPy-based direct-current generator. Output currents (left panel), output voltages (middle panel) of direct-current generators: Au/PPy/Al, Au/PPy/Ag, Au/PPy/Cu, Au/PPy/Pt (4% compressive deformation), respectively. The relationship of the output currents/voltages with varied contacted metals (right panel).

**Figure. S8** Output characteristics of Al/PPy/Au generator under different deformations. Output current (left panel) and voltage (middle panel) of Al/PPy/Au generator during variation degree of compressing deformation and corresponding extracted the relationships of current ~ compressing deformation and voltage ~ compressing deformation (right panel).

**Figure. S9** Output characteristics of Ag/PEDOT/Au generator under different deformations. Output current (left panel), voltage (middle panel) of Ag/PEDOT/Au generator during variation degree of compressing deformation and corresponding extracted the relationships of current ~ compressing deformation and voltage ~ compressing deformation (right panel).

**Figure. S10** Output characteristics of Ag/PANI/Cu generator under different deformations. Output current (left panel), voltage (middle panel) of Ag/PANI/Cu generator during variation degree of compressing deformation andcorresponding extracted the relationships of current ~ compressing deformation and voltage ~ compressing deformation (right panel).
